# Urinary Dysfunction: Frequency, Risk factors, and Interventions in patients with Cancer during Acute Inpatient Rehabilitation

**DOI:** 10.7150/jca.80620

**Published:** 2023-01-09

**Authors:** Jegy Mary Tennison, Annie Pally, Bryan Michael Fellman, Ouida Lenaine Westney, Eduardo Bruera

**Affiliations:** 1 Section of Physical Medicine & Rehabilitation, Department of Palliative, Rehabilitation, and Integrative Medicine, The University of Texas MD Anderson Cancer Center, Houston, Texas, USA.; 2 Department of Neurology, Dell Medical School, The University of Texas at Austin, Austin, Texas, USA.; 3 Department of Biostatistics, The University of Texas MD Anderson Cancer Center, Houston, Texas, USA.; 4 Department of Urology, The University of Texas MD Anderson Cancer Center, Houston, Texas, USA.; 5 Section of Palliative Medicine, Department of Palliative, Rehabilitation, and Integrative Medicine, The University of Texas MD Anderson Cancer Center, Houston, Texas, USA.

**Keywords:** risk factors, inpatient, neoplasms, lower urinary tract symptoms, urinary disorders, pelvic floor

## Abstract

**Introduction:** Urinary dysfunction has a strong impact clinically, socially, and economically. Although the development of acute urinary dysfunction in hospitalized patients with cancer is common in clinical practice, its occurrence and management strategies are scant in the literature. It has been reported as one of the more common medical complications in patients with cancer undergoing acute inpatient rehabilitation. This study assessed the frequency of and risk factors for acute urinary dysfunction among these patients and identified the interventions used for management.

**Methods:** This is a retrospective study of consecutive patients admitted to a National Cancer Institute Comprehensive Cancer Center's acute inpatient rehabilitation service from 9/1/2020 through 3/15/2021. We excluded patients that were readmissions during the study time frame. We collected patients' demographic, clinical, and functional data. We defined acute urinary dysfunction as the development of any new urinary symptom(s) or diagnosis, which involved additional work-up and/or management after admission to the acute inpatient rehabilitation service.

**Results:** Of the 176 total patients included in this study, 47 (27%; 95% confidence interval [CI], 20-34) patients had acute urinary dysfunction. The most frequent diagnoses were urinary tract infection (32%) and neurogenic bladder (26%). The most common tests were urine cultures (32%) and urinalyses (30%). The most commonly prescribed medications were antibiotics (32%) and alpha-1 blockers (15%). Other most frequent interventions included timed voiding (34%) and intermittent catheterization with bladder scans (28%). Acute urinary dysfunction was associated with an increased length of stay on the inpatient rehabilitation service (odds ratio [OR], 1.13; 95% CI, 1.06-1.20; *P*<.001), surgery during the index admission (OR, 2.50; 95% CI, 1.21-5.16; *P*=.014), and fecal incontinence (OR, 6.41; 95% CI, 1.83-22.44; *P*=.004).

**Conclusion:** Acute urinary dysfunction was noted to be a substantial problem in this cohort. This is an overlooked dimension of inpatient cancer rehabilitation that deserves more attention. Patients at risk for acute urinary dysfunction may benefit from close monitoring for medical management and rehabilitation interventions to maximize functional independence with bladder care. More research regarding acute urinary dysfunction types and management approaches in post-acute care settings for patients with cancer is justified.

## Introduction

Urinary dysfunction can manifest with symptoms such as incontinence, nocturia, urgency, frequency, and urinary retention 1. These symptoms adversely affect sleep, mood, the ability to perform the activities of daily living 2, and quality of life 3. Urge urinary incontinence has been rated as the most bothersome symptom of urinary dysfunction among both women and men 4 and is associated with a substantial economic burden in the United States 5. Neurogenic lower urinary tract dysfunction has been shown to be a substantial economic burden for healthcare systems worldwide 6.

Patients with cancer can develop urinary dysfunction due to their disease 7 or its treatments, such as chemotherapy, immunotherapy, pelvic radiation, or surgery 7,8. Previous studies on urinary dysfunction primarily concentrated on a few specific cancer types and a variety of urinary dysfunction types 9-12. Patients with cancer undergoing acute inpatient rehabilitation after hospitalization, however, have a wide variety of heterogeneous cancer types. The purpose of acute inpatient rehabilitation is to provide an intensive and a total of three hours of rehabilitation sessions per day at least five days of the week generally (or a total of fifteen hours of rehabilitation sessions over consecutive seven days) while under the management of a rehabilitation physician with weekly interdisciplinary team meetings. These sessions require the need of at least two or more therapies (i.e. physical, occupational, speech, etc.) in stable patients who can actively participate and have measurable functional improvements.

In the practice of physical medicine and rehabilitation, urinary dysfunction is often due to neurological conditions affecting the brain and spinal cord 13. These conditions can lead to morbid urological complications, such as urinary tract infections, urinary incontinence, urolithiasis 13,14, urosepsis, ureteric obstruction, vesicoureteral reflux, and renal failure 14.

Neurological deficits and deconditioning due to cancer are common triggers for the need for acute inpatient rehabilitation 15 following hospitalization. One study assessing medical complications among patients with cancer undergoing acute inpatient rehabilitation found that 38% had genitourinary or renal disorders 16. To our knowledge, however, acute urinary dysfunction types, occurrences, and management strategies among patients with cancer undergoing acute inpatient rehabilitation have not been studied. Therefore, this study assessed the frequency of and risk factors for acute urinary dysfunction among these patients and identified the interventions used to manage the condition. Research data in this area is essential to improving healthcare quality, especially concerning the comprehensive and interdisciplinary medical and rehabilitative management approaches.

## Methods

### Subjects, selection criteria, and data source

This retrospective study was conducted with approval from the Institutional Review Board. Data were collected from the institutional electronic health records and the acute inpatient rehabilitation admissions log. The data were managed using Research Electronic Data Capture software (REDCap 12.5.9 - © 2022 Vanderbilt University). The patients admitted to M. D. Anderson's acute inpatient rehabilitation service have to be of 18 years or older and have a primary rehabilitation impairment that was associated with their cancer diagnosis or treatment-related hospital admission. This study initially comprised 181 patients with cancer who were consecutive admissions to the acute inpatient rehabilitation service from September 1, 2020, through March 15, 2021. It then excluded five patients who were readmitted again to the rehabilitation service during the study period of September 1, 2020, through March 15, 2021, to avoid duplicating patient medical record numbers. This resulted in a total cohort of 176 unique patient admissions to evaluate for this study. (It did include patients who might have had another acute inpatient rehabilitation stay before the study period since they would have unique medical record numbers to include within the study time frame).

### Data reviewed

Data collected at the time of admission for acute inpatient rehabilitation included demographic information (age, sex, race, and ethnicity) and clinical characteristics (primary neoplasm type, occurrence of surgery during the index admission). We reviewed daily rehabilitation progress notes and discharge summaries to obtain additional clinical characteristics (length of stay for acute inpatient rehabilitation, severity of cognitive deficits as determined by speech and language pathologists, presence or absence of fecal incontinence, and the urinary dysfunction details).

Functional status was measured using the Activity Measure for Post-Acute Care (AM-PAC) Inpatient “Six Clicks” Short Forms, which are validated 17 and reliable 18. A physical therapist completed the AM-PAC basic mobility form, and an occupational therapist completed the AM-PAC daily activity form within 24 hours after admission and 24 hours before discharge from the acute inpatient rehabilitation service.

### Subgroups

We defined acute urinary dysfunction as the development of any new urinary symptom(s) or diagnosis requiring work-up and/or management after admission to the acute inpatient rehabilitation service. Thus, any urinary dysfunction already present upon admission to the acute inpatient rehabilitation service was considered an established urinary dysfunction. We divided the total cohort into 2 groups for comparison: 1) patients with acute urinary dysfunction and 2) patients without acute urinary dysfunction (no new urinary dysfunction *or* established urinary dysfunction without any new urinary dysfunction).

### Statistical analyses

The demographic and clinical characteristics of the study population were summarized using standard summary statistics such as medians, interquartile percentages, and ranges for continuous variables and frequencies and percentages for categorical variables. Patient characteristics were compared according to the presence or absence of acute urinary dysfunction using a *t*-test or Wilcoxon rank-sum test for continuous variables and a chi-squared or Fisher's exact test for categorical variables. We estimated acute and established urinary dysfunction and established 95% confidence intervals (CIs) to determine proportions and compare them. Types of urinary dysfunction were described with frequency percentages. Logistic regression models were conducted to estimate the odds of acute urinary dysfunction based on clinical, functional, and demographic variables.

## Results

Of the 176 patients included in this study, 47 (27%; 95% CI, 20-34) patients had acute urinary dysfunction during acute inpatient rehabilitation. Table 1 provides an overview of the demographic information, clinical characteristics, and functional scores for the total cohort and the subgroups with and without acute urinary dysfunction. Of note and for comparison, there were 29 (16%; 95% CI, 11-23) patients with an established urinary dysfunction. In the total cohort of 176 patients, most patients were males (59%), White (83%), and had undergone surgery (57%) during the index admission. The median age was 67, and the median length of stay on the acute inpatient rehabilitation service was 10 days. The primary neoplasm types were hematologic and lymphatic (26%) and brain and other nervous systems (21%). The acute inpatient rehabilitation length of stay was higher in the acute urinary dysfunction group (median 11 days vs. 10 days; p = 0.002). There was a higher proportion of surgery during hospitalization in the acute urinary dysfunction group (72% vs. 51%; p = 0.012). There was also a higher proportion of fecal incontinence during acute inpatient rehabilitation in the acute urinary dysfunction group (17% vs. 3%; p = 0.003). The other variables in Table 1 were not statistically significant between the two groups (acute urinary dysfunction and no acute urinary dysfunction groups).

Table 2 describes the characteristics of acute urinary dysfunction with frequencies and percentages. This study had only single (not multiple) acute urinary dysfunction diagnoses per patient. The most frequent diagnoses were urinary tract infection (32%) and neurogenic bladder (26%). The most common tests were urine cultures (32%) and urinalyses (30%). The most commonly prescribed medications were antibiotics (32%) and alpha-1 blockers (15%). Other most frequent interventions included timed voiding (34%) and intermittent catheterization with bladder scans (28%).

Table 3 provides the univariate logistic regression estimates of the odds that a particular event was associated with acute urinary dysfunction. As the acute inpatient rehabilitation length of stay increased, the odds of having acute urinary dysfunction increased (OR: 1.13; 95% CI, 1.06 - 1.20; p<.001). Those who had surgery during hospitalization had 2.50 (95% CI, 1.21 - 5.16; p = .014) times the odds of having acute urinary dysfunction. Those with fecal incontinence during acute inpatient rehabilitation had 6.41 (95% CI, 1.83 - 22.44; p = .004) times the odds of having acute urinary dysfunction. The other variables in Table 3 were not statistically significant.

## Discussion

In this cohort, we found a significant rate (27%) of acute urinary dysfunction among patients with cancer undergoing acute inpatient rehabilitation. Previous studies on urinary dysfunction in patients with cancer reported a variety of urinary dysfunction types affecting the quality of life 9-12. These studies concentrated on patients with specific cancer types such as prostate, colorectal, endometrial, and ovarian cancer 9-12. Other studies have described urinary dysfunction primarily in patients undergoing inpatient rehabilitation for strokes 19, brain disorders 20-21, spinal cord disorders 22-24, and other neurological conditions. To our knowledge, this is the first study to report the rate of acute urinary dysfunction among patients with cancer and undergoing acute inpatient rehabilitation. Identifying acute urinary dysfunction in these patients is essential because urinary symptoms can affect patients' participation in rehabilitation programs 25. Improving patients' ability to independently manage their bladder (and bowel) function is a fundamental goal of acute inpatient rehabilitation, in addition to improving patients' physical functioning levels. If patients cannot manage their bladders adequately, urological complications can occur. Urological complications such as urolithiasis and renal failure have been reported to cause hospital readmissions shortly after discharge from acute inpatient cancer rehabilitation, increasing the 30-day readmission rate 26. Of note, acute renal insufficiency or failure was our study's third most frequent type of acute urinary dysfunction.

Patients with cancer undergoing acute inpatient rehabilitation have a variety of impairments for which rehabilitation is needed (e.g., debility/deconditioning, medically complex conditions, and, in patients needing neurorehabilitation, brain and/or spinal cord dysfunction is common) 27. This difference in case mix can account for the varying types of urinary dysfunction these patients experience such as urinary tract infection, neurogenic bladder, and other (see Table 2) wide variety of urinary dysfunctions. Patients with cancer are often transferred to the acute inpatient rehabilitation service with an external urethral catheter in place, and removal of the urethral catheter during acute inpatient rehabilitation may lead to a new diagnosis of acute urinary dysfunction. Patients undergoing neurorehabilitation are at increased risk for significant urological complications due to neurological conditions that may be severe and/or permanent. For example, in our patient cohort, the 12 individuals diagnosed with a neurogenic bladder included 8 patients who had undergone spinal surgery for metastatic spinal cord compression; 1 who had undergone a sacral chordoma resection; 1 who had undergone a hemipelvectomy due to osteosarcoma; 1 with pelvic sarcoma; and 1 with bladder detrusor underactivity.

The most frequent diagnoses in our cohort were urinary tract infection (32%) and neurogenic bladder (26%), both of which are also common in patients with spinal cord injuries 28. Urinary tract infections are a common source of morbidity among patients undergoing rehabilitation for spinal cord injuries 23. Moreover, studies of patients undergoing neurorehabilitation for stroke, brain, and spinal cord disorders have demonstrated that decreased functional scores are associated with urinary tract infections 29-31. In our study, there was no statistical significance in the functional scores between the patients with and without acute urinary dysfunction (see Table 1). This may be related to the fact that our patients were treated in an acute care hospital in which diagnosis and management are rapid and resources for investigations and consultations are readily available; these facility characteristics improve patients' chances of achieving maximal functional recovery. Access to this level of care may not be available at free-standing rehabilitation facilities physically disconnected from a hospital.

The most frequently ordered tests for the development of new urinary symptoms were urine cultures (32%) and urinalyses (30%); the most commonly prescribed medications were antibiotics (32%) and alpha-1 blockers (15%). These are appropriate tests and medications for this study's most frequently identified urinary dysfunction diagnoses. The management of urinary dysfunction includes interventions such as education, lifestyle advice 32, facilitative techniques, rehabilitation, catheterizations, and pharmacologic treatment 13 (as noted in our study in Table 2), and surgery may also be required 13. In our study population, no surgical intervention was warranted, even for patients who required urology consultations. Specialists (from the departments of urology, nephrology, infectious diseases, and internal medicine) were consulted in 7 (15%) cases. These findings highlight the need for possible multimodal and interdisciplinary approaches to managing acute urinary dysfunction.

The factors associated with acute urinary dysfunction were surgery during the index admission, a longer inpatient rehabilitation length of stay, and fecal incontinence during acute inpatient rehabilitation. Surgery has been known to be associated with symptoms of urinary dysfunction, such as urinary retention 33 and urinary tract infections 34. Postoperative urinary tract infections have been associated with longer hospital stays 35. Finally, the clinical comorbidity between bladder and bowel dysfunction has been demonstrated in many reports 36. There are many correlations between the lower urinary and gastrointestinal tracts, such as their embryological origins, anatomical positions in the pelvis, use of the same supporting muscles, joint peripheral innervation and coordination of viscera, and similar functions of storing and evacuating waste 36.

### Strengths and limitations of the study

This small, retrospective study was conducted at a single institution in a full-service academic medical center to describe the characteristics of acute urinary dysfunction in the specific setting of acute inpatient cancer rehabilitation. Retrospective by nature would depend on the data available to collect and analyze. Thus, other unaccounted risk factors could have confounded the results. The generalizability of our findings to other facilities' acute inpatient cancer rehabilitation services may be limited owing to the differences in referral patterns to an acute inpatient rehabilitation facility.

Despite these limitations, this study fills a knowledge gap and highlights the importance of assessing for acute urinary dysfunction among patients with cancer undergoing inpatient rehabilitation. The information it provides regarding the nature and management of acute urinary dysfunction in patients with cancer can be helpful to clinicians and consultants involved in rehabilitation settings providing comprehensive and interdisciplinary care. The data can also be used to convince payors to authorize the transfer of medically complex patients, such as these patients with cancer at risk for urinary dysfunction, to acute inpatient rehabilitation as opposed to subacute rehabilitation settings (i.e., skilled nursing facilities, long-term acute care hospitals), in which resources for managing urological complications may be limited.

## Conclusions

In this study, we found acute urinary dysfunction among more than a quarter of the patients with cancer undergoing acute inpatient rehabilitation. Acute urinary dysfunction was associated with a longer inpatient rehabilitation length of stay, surgery during the index admission, and fecal incontinence. There should be a low threshold of suspicion for acute urinary dysfunction in patients with cancer after hospitalization and admitted for acute inpatient rehabilitation. Urinary symptoms should be closely monitored for medical and symptom management and to improve patients' independence with bladder care. Further research regarding acute urinary dysfunction in patients with cancer in other settings globally is needed to understand the nature, frequency, and management patterns.

## Figures and Tables

**Table 1:** Comparison of patients with and without acute urinary dysfunction during inpatient cancer rehabilitation

Characteristics	Total, n = 176	Acute urinary dysfunction, n = 47	Established/no new urinary dysfunction, n = 129	*P*-value^a^
**Sex, n (%)**				.864^b^
Male	103 (59)	28 (60)	75 (58)	
Female	73 (41)	19 (40)	54 (42)	
**Race, n (%)**				.683^c^
White	146 (83)	38 (81)	108 (84)	
Black	15 (8)	5 (10)	10 (8)	
Asian	12 (7)	4 (9)	8 (6)	
Others	3 (2)	0 (0)	3 (2)	
**Ethnicity, n (%)**				.502^b^
Hispanic	18 (10)	6 (13)	12 (9)	
Non-Hispanic	158 (90)	41 (87)	117 (91)	
**Primary neoplasm type, n (%)**				.075^c^
Hematologic and lymphatic	45 (26)	9 (19)	36 (28)	
Brain and other nervous systems	37 (21)	10 (21)	27 (21)	
Genitourinary system	20 (11)	8 (17)	12 (9)	
Digestive system	15 (9)	4 (9)	11 (9)	
Respiratory system	12 (7)	5 (11)	7 (5)	
Bone and connective tissue	10 (6)	6 (13)	4 (3)	
Oral cavity and pharyngeal	10 (6)	1 (2)	9 (7)	
Others^d^	27 (15)	4 (9)	23 (18)	
**Surgery during the index admission, n (%)**				***.012^b^***
Yes	100 (57)	34 (72)	66 (51)	
No	76 (43)	13 (28)	63 (49)	
**Fecal incontinence, n (%)**				***.003* ^c^**
Yes	12 (7)	8 (17)	4 (3)	
No	164 (93)	39 (83)	125 (97)	
**Cognitive deficits, n (%)**				.077^ c^
None	133 (76)	33 (70)	100 (77)	
Mild	25 (14)	7 (15)	18 (14)	
Moderate	13 (7)	3 (6)	10 (8)	
Severe	5 (3)	4 (9)	1 (1)	
Age in years, median (IQR^e^)	67 (56, 73)	65 (53, 70)	68 (56, 73)	.092^f^
Acute inpatient rehabilitation length of stay in days, median (IQR)	10 (7, 14)	11 (9, 16)	10 (7, 13)	***.002* ^ f^**
AM-PAC^d^ Basic Mobility score at admission, median (IQR)	41 (38, 44)	41 (35, 44)	41 (39, 44)	.203^f^
AM-PAC Daily Activity score at admission, median (IQR)	37 (35, 40)	36 (33, 39)	37 (35, 40)	.071^f^
AM-PAC Basic Mobility score at discharge, median (IQR)	44 (41, 50)	44 (39, 48)	44 (41, 50)	.094^f^
AM-PAC Daily Activity score at discharge, median (IQR)	40 (37, 42)	40 (36, 42)	40 (38, 42)	.371^f^

^a^ Boldface indicates a statistically significant difference; ^b^ Chi-squared test; ^c^ Fisher's exact test; ^d^ Other neoplasms included breast, endocrine, eye and orbit, skin, thymus, other soft tissue, and unspecified primary site; ^e^ IQR = interquartile range; ^f^ Wilcoxon rank-sum test; ^g^ AM-PAC = Activity Measure for Post-Acute Care (Inpatient “Six Clicks” Short Form).

**Table 2:** Acute urinary dysfunction characteristics among 47 patients with cancer undergoing inpatient rehabilitation^a^

Presence of acute urinary symptoms	N = 47, (100%)
**Diagnosis/symptoms**	
Urinary tract infection	15 (32)
Neurogenic bladder	12 (26)
Acute renal insufficiency/failure	9 (19)
Dysuria/urinary retention that improved	3 (6)
Benign prostate hypertrophy exacerbation	2 (4)
Functional incontinence	2 (4)
Pelvic floor dysfunction	2 (4)
Post-operative urinary retention	1 (2)
Urinary frequency with negative work-up	1 (2)
***Interventions***	
**Test**	
Urine culture	15 (32)
Urinalysis	14 (30)
Renal function test	6 (13)
Renal ultrasound	1 (2)
**Medication**	
Antibiotic for urinary tract infection	15 (32)
Alpha-1 blocker	7 (15)
Intravenous hydration	5 (11)
5-alpha-reductase inhibitor	1 (2)
Anticholinergic agent	1 (2)
Phenazopyridine	1 (2)
**Consultation**	
Nephrology	3 (6)
Urology	2 (4)
Infectious diseases	1 (2)
Internal medicine	1 (2)
**Procedure**	
Nephrostomy tube removal	1 (2)
**Other**	
Timed voiding	16 (34)
Intermittent catheterization with bladder scans	13 (28)
Urethral catheter	8 (17)
Condom catheter	1 (2)
Facilitative techniques and maneuvers (massage, double voiding, standing)	6 (13)
Education/lifestyle advice	5 (11)
Pelvic rehabilitation/exercise	1 (2)

^a^ Total cohort = 176 patients.

**Table 3:** Univariate logistic regression estimates of the odds of acute urinary dysfunction

Characteristic^a^	Odds ratio	95% CI	*P*-value^b^
**Age**	0.98	0.96 - 1.01	.141
**Sex**			
Female	1.00	1.00	
Male	1.06	0.54 - 2.09	.864
**Race**			
White	1.00	1.00	
Other^c^	1.22	0.51 - 2.89	.655
**Ethnicity**			
Hispanic	1.00	1.00	
Not Hispanic	0.70	0.25 - 1.99	.504
Inpatient rehabilitation length of stay	1.13	1.06 - 1.20	***< .001***
**Primary neoplasm diagnosis**			
Bone and connective tissue	1.00	1.00	.104
Brain and other nervous systems	0.25	0.06 - 1.06	
Digestive system	0.24	0.04 - 1.33	
Hematologic and lymphatic	0.17	0.04 - 0.72	
Oral cavity and pharyngeal	0.07	0.01 - 0.84	
Respiratory system	0.48	0.09 - 2.63	
Genitourinary system	0.44	0.09 - 2.09	
Others^d^	0.12	0.02 - 0.60	
**Surgery during the index admission**			
No	1.00	1.00	
Yes	2.50	1.21 - 5.16	***.014***
**Cognitive dysfunction during rehab stay**			
No	1.00	1.00	
Yes	1.46	1.69 - 3.10	.320
**Level of cognitive dysfunction**			
Mild	1.00	1.00	.177
Moderate	0.77	0.16 - 3.66	
Severe	10.29	0.97 - 108.81	
None	0.85	0.33 - 2.21	
**Fecal incontinence during rehab stay**			
No	1.00	1.00	
Yes	6.41	1.83 - 22.44	***.004***
**AM-PAC^e^ Basic Mobility score at admission**	0.96	0.91 - 1.02	.174
**AM-PAC Daily Activity score at admission**	0.99	0.93 - 1.05	.649
**AM-PAC Basic Mobility score at discharge**	0.96	0.91 - 1.01	.141
**AM-PAC Daily Activity score at discharge**	0.98	0.91 - 1.04	.441

^a^ Characteristics were assessed based on a series of univariate logistic regression models with factor significance determined by the Wald Chi-Square Test; ^b^ Boldface indicates a statistically significant difference; ^c^ Black and Asian were combined with “Other” to make the estimation more reliable because there were few individuals in these categories; ^d^ Other neoplasms included breast, endocrine, eye and orbit, skin, thymus, other soft tissue, and unspecified primary site cancers; ^e^ AM-PAC = Activity Measure for Post-Acute Care (Inpatient "Six Clicks" Short Form).

## Abbreviations

AM-PAC: Activity Measure for Post-Acute Care
CI: confidence intervals
